# Current Welfare Problems Facing Horses in Great Britain as Identified by Equine Stakeholders

**DOI:** 10.1371/journal.pone.0160269

**Published:** 2016-08-08

**Authors:** Susan V. Horseman, Henry Buller, Siobhan Mullan, Helen R. Whay

**Affiliations:** 1 School of Veterinary Sciences, University of Bristol, Bristol, England; 2 Department of Geography, University of Exeter, Exeter, England; University of Sydney, AUSTRALIA

## Abstract

Despite growing concerns about the welfare of horses in Great Britain (GB) there has been little surveillance of the welfare status of the horse population. Consequently we have limited knowledge of the range of welfare problems experienced by horses in GB and the situations in which poor welfare occurs. Thirty-one in-depth interviews were conducted with a cross -section of equine stakeholders, in order to explore their perceptions of the welfare problems faced by horses in GB. Welfare problems relating to health, management and riding and training were identified, including horses being under or over weight, stabling 24 hours a day and the inappropriate use of training aids. The interviewees also discussed broader contexts in which they perceived that welfare was compromised. The most commonly discussed context was where horses are kept in unsuitable environments, for example environments with poor grazing. The racing industry and travellers horses were identified as areas of the industry where horse welfare was particularly vulnerable to compromise. Lack of knowledge and financial constraints were perceived to be the root cause of poor welfare by many interviewees. The findings give insight into the range of welfare problems that may be faced by horses in GB, the contexts in which these may occur and their possible causes. Many of the problems identified by the interviewees have undergone limited scientific investigation pointing to areas where further research is likely to be necessary for welfare improvement. The large number of issues identified suggests that some form of prioritisation may be necessary to target research and resources effectively.

## Introduction

Estimates suggest that there are at least one million horses and ponies in Great Britain (GB) [1], the majority of which are kept for sport and leisure purposes [2]. In recent years concern has grown that the welfare of many of these horses may be suboptimal. Two consecutive reports, published collaboratively by the main UK based equine welfare charities, highlight these concerns and outline fears that the welfare charities will soon have insufficient resources to cope with the number of horses needing their assistance [3, 4]. However, there has been limited empirical surveillance of the welfare of horses in GB and surveillance which has occurred has focused on equine health issues over other aspects of welfare. For example, Wylie et al [5] investigated the prevalence of laminitis within veterinary registered horses in GB, Murray et al [6] collected survey data on lameness prevalence in dressage horses in the UK and Ireland et al [7] investigated health in geriatric horses in the UK. A more holistic approach was taken by Samual et al [8] and Mullan et al [9] who investigated all aspects of the welfare of tethered and free ranging horses on common land in South Wales. However, until recently, holistic assessment protocols which integrate the many facets of welfare have been lacking for horses [10] and no surveillance of all aspects of welfare across the GB equine population has been conducted. We therefore may have incomplete knowledge of the range of welfare problems faced by the GB horse population as a whole, limiting our ability to improve the situation.

It is becoming increasingly common to capture data about animal welfare through qualitative research approaches, especially where there are limited objective welfare assessment tools available [11]. Stakeholders, for example companion animal owners, farmers, horse owners and veterinary surgeons can have direct experience of the welfare issues which exist and consultation of stakeholders has been used to identify welfare problems in companion animals (for example [11]; dogs), farm animals (for example [12]; dairy cows) and equine species (for example [13]; working equids). Collins et al [14, 15] utilised a Delphi approach, whereby experts were consulted in three systematic, iterative rounds, to facilitate the identification of the main welfare problems facing horses in Ireland. Their research identified horse disposal and informal gatherings, for example horse fairs, as areas where horse welfare in Ireland is particularly vulnerable to compromise. These findings were then used to inform further investigation [16]. Conducting similar research is likely to facilitate the identification of the current welfare problems facing horses in GB.

The aim of the research presented was to explore equine stakeholders’ perceptions of the welfare problems affecting horses in GB. The focus of the research was on those who, either through work or pleasure, come into daily contact with horses. To reflect the diversity of the equine industry, the authors aimed to consult a range of stakeholders involved with a spectrum of horse activities or uses and who held a variety of roles. In-depth, semi-structured interviews were used as this approach allows subjects to bring up topics which the researcher may not have considered [17] and as such is particularly appropriate to use when little is known about a subject area. Kauppinen et al [18] and Horseman et al [19] found that by using in-depth interviews in their animal welfare research, new themes emerged, relating to their area of interest, which had not been highlighted in previous research. In the current study this approach was used to ensure that the research frame was not limited based on the researchers’ preconceptions. Through open-ended questioning the authors aimed to explore the range of welfare problems which may exist in GB without being constrained by the existing literature or their own ideas.

## Materials and Methods

The below methods were carried out in accordance with University of Bristol ethical guidance and ethical approval was sought and granted by the University of Bristol’s ethics committee before commencement.

### Recruitment

Population data reported by Boden et al [2] was used, along with the research teams’ first- hand knowledge of the equine industry, to inform participant recruitment. The authors aimed to consult a broad cross-section of stakeholders considering the range of activities that horses engage in (as outlined by [2]) and the different ways in which stakeholders are involved with the industry. However, a statistically representative sample was not sought. Four distinct categories of stakeholder involvement were identified: equine health, riding/training, welfare charity/enforcement work and leisure use (see Table 1 for full description of each category). Interviewees were recruited across all four of these categories and a cross-section of disciplines including leisure riding, show jumping, dressage, eventing and racing.

**Table 1:** Description of the four categories of involvement that the interviewees fell into and details of the specific roles that the interviewees held at the time of interview.

Category	Description of category	Role of interviewees at time of interview
Equine health	Interviewees in this category were engaged in activities that promoted equine health/physical well- being or had roles associated with the end of horses’ lives	• **Vet x 2**, provided veterinary treatment mainly to leisure horses • **Farrier x 2**, provided services trimming and shoeing horses’ feet • **Equine podiatrist**, provided services trimming horses’ feet • **McTimoney chiropractic practitioner**, provided complimentary musculo-skeletal treatments to horses • **Abattoir owner**, provided services killing horses for meat to enter the human food chain • **Knackerman**, provided services in killing and disposing of horses that could not enter the human food chain
Riding/Training	Interviewees in this category were mainly engaged in activities around riding horses, training horses and/or training riders	• **Dressage trainer and rider**, provided services training dressage horses and riders • **Show jumping trainer**, provided services training show jumping horses and riders • **Show pony owner**, involved in breeding and producing show ponies • **Driving coach**, provided services training horses and drivers for carriage driving • **Point to point rider**, rode horses in amateur jump races • **Race trainer**, provided training for jump race horses • **Rehabilitation yard owner**, provided services retraining horses with physical or behavioural problems • **Polo player and event rider,** involved in breeding and producing polo ponies and competed in polo and eventing • **Endurance rider,** involved in endurance riding at an international level
Equine welfare charity/enforcement	Interviewees in this category held roles within equine charities and/or were involved with enforcing welfare legislation	• **Senior equine welfare charity worker**, involved in co-ordinating responses to equine welfare complaints and equine welfare education • **Welfare charity centre manager,** responsible for running a large equine rescue/ rehoming centre • **Equine welfare charity centre groom,** responsible for the day to day care and rehabilitation of rescued horses • **Equine welfare charity field officer x 2,** responsible for investigating welfare complaints made to welfare charities • **Trading standards officer,** government officer involved in enforcing animal welfare legislation including that relevant to horses
Leisure use	Interviewees in this category were mainly involved with horses kept for leisure purposes	• **Leisure horse owner x 2,** owned horses for leisure purposes • **Leisure horse loaner,** loaned a horse from another horse owner for leisure purposes • **Livery yard owner**, responsible for providing stabling, grazing and care for other peoples’ leisure horses in return for a fee • **Semi-feral pony owner,** owner of ponies that lived on the New Forest • **Member of the travelling community,** that owned and bred leisure horses • **Owner of a retired leisure horseFreelance instructor and groom,** taught riding and looked after other peoples’ leisure horses on a self-employed basis

Opportunistic sampling and snow- balling techniques were employed. Snow- balling is a recruitment method whereby those already recruited are asked to nominate others known to them who may also be suitable recruitees [20]. The authors recognise that this approach may have introduced bias into the study.

Initial recruitment was carried out by telephone by the first author, using contacts known to the authors and wider research team. The author introduced themselves and gave potential recruitees background information about the aims and objectives of the research, before asking if they would be willing to be interviewed as part of the study. Where recruitment was successful a time and date was arranged for the face to face interview and verbal permission to audio record the interview sought. All of the potential recruits contacted agreed to take part in the study. One person who was initially recruited later removed themselves from the study prior to interview, due to work commitments. An individual holding a similar role to the person who withdrew was subsequently recruited.

### Data Collection

Informed by the researchers’ areas of interest, an interview outline was devised consisting of topics to be covered in the interviews. The broad topics for discussion in all of the interviews were: 1) How ‘equine welfare’ is defined when a direct question is asked, 2) exploration of what horses need to ensure their welfare, 3) exploration of what may result in a horse having poor welfare, including the conditions which may lead to poor welfare, and 4) exploration of examples of poor welfare witnessed by the interviewees. A pilot interview was conducted by the first author, with a leisure horse owner, to ensure that the topics chosen allowed for exploration of the areas of interest, and that the line of questioning was likely to be acceptable to the interviewees. Feedback from the interviewee and the interviewer’s observations confirmed that the interview structure was fit for purpose and no changes were made as a result of the pilot. It was noted by the authors that piloting across a greater range of stakeholders may have allowed more certainty around the ‘acceptability’ of the line of questioning. However, it was considered difficult to predict where differences in perceptions of ‘acceptability’ may lie. As such a single pilot was considered sufficient on the proviso that the interviewees were informed of their right to refuse to answer any questions at the beginning of the interview, and that the interviewer would moderate the line of questioning if the interviewee appeared uncomfortable at any point.

The interviews were carried out at a location of the interviewees’ choice, usually their home or the yard at which they worked or kept their horse, and lasted between half an hour and two hours, dependent on the amount of detail given in the interviewees’ responses. All of the interviews were conducted by the first author to ensure consistency and were audio recorded. The interviewer was a PhD student with prior experience of conducting in-depth interviews. Before the interviews began the interviewees were reminded of the purpose of the study and were asked to sign a consent form in accordance with University of Bristol ethical guidance. Interviewees were assured that their responses would be anonymised, and that the data collected would be stored securely and destroyed at the end of the study. They were also informed that they did not have to answer any question which they felt uncomfortable answering. The interviewer asked the interviewees to provide information about their background, particularly in relation to their prior experience with horses, before asking questions around the four areas described above. These topics were then fully explored using follow up questions which were driven by the interviewees’ responses. For example, where a particular management practice was brought up by an interviewee as a cause of poor welfare in GB, the interviewer would follow up with questions to explore the specific welfare consequences of the practice and how common the interviewee believed the practice was.

### Data analysis

The interview recordings were transcribed (*verbatim*) and the resulting transcripts were analysed by the first author. Firstly, themes and sub-themes which related specifically to discussions about poor equine welfare in GB, as witnessed by the interviewees, were identified using a qualitative analysis approach [17]. The transcripts then underwent content analysis [17] to identify the specific welfare issues discussed within each of the themes. For example, within the recognised theme of ‘welfare problems faced by horses in GB’ a sub theme of ‘health related welfare problems’ was identified incorporating the specific welfare issue of ‘laminitis’. The interviewees were grouped and assigned to one of the four categories (equine health, riding/training, welfare charity/enforcement work and leisure use) based on their primary roles and areas of knowledge at the time of the interview (see Table 1). The specific issues brought up by each of the interviewees were recorded and analysed using SPSS v 21 for Windows (SPSS Inc, USA). Descriptive statistics relating to the number of interviewees discussing each theme and issues were produced and 2-sided Fisher’s Exact Tests were conducted to look for associations between the category of the interviewees’ involvement and the issues they brought up in their interviews.

As the interviewees did not always label the examples they were giving specifically as examples of poor ‘welfare’ and sometimes used alternative language, responses were included if the interviewees discussed an issue as having some form of negative impact on horses in GB. For example, some interviewees discussed how horses in GB were sometimes stabled 24 hours a day (24/7) having negative effects on horses in GB. However, they did not always directly refer to stabling 24/7 as a ‘welfare’ problem, for example when it was carried out as a means of protecting the horse’s health (see [21] for full discussion).

### Responses

Thirty one interviews were conducted to ensure coverage across the main stakeholder roles and disciplines but not every possible combination. Table 1 gives a description of each of the interviewee categories, the interviewees within each category and details of their involvements with the equine industry at the time of the interviews. Other demographic information, for example the age, gender and years of equine experience of the interviewees, is not referred to as these data were either not consistently collected as a result of the methodology and/or not incorporated into the reported analysis. Whilst the authors recognise that some demographic features, for example, the number of years of experience the respondents had with horses and the number of horses the respondents came into contact with may have impacted on their perceptions, such consideration was beyond the scope of this research.

## Results

Through thematic analysis four themes associated with the responses relating to examples of poor equine welfare in GB, the geographical area of interest, were identified: i) The welfare problems that are experienced by horses, including sub themes of health, management and riding and training problems. These were specific, individual welfare issues or else specific conditions or practices that were perceived to cause poor welfare in horses in GB, for example laminitis and tethering. ii) The contexts in which horse welfare is compromised in GB. These were situations where a number of specific welfare issues or risk factors for poor welfare were perceived to be present, for example contexts where horses are transported. iii) The specific uses of horses/disciplines in which horses are used where welfare problems occur in GB and iv) the root causes of poor horse welfare in GB. These were more fundamental underlying root causes of poor welfare that were seen to underpin practices relating to horse welfare, for example lack of knowledge on the part of caregivers. These themes are further described below. It should be noted that the interviewees were not prompted to discuss specific welfare issues or practices associated with welfare and as such all welfare issues discussed by the interviewees were freely raised by the participants.

### Welfare problems experienced by horses

During the interviews stakeholders discussed specific welfare problems they perceived to affect horses in GB. These perceptions were based largely on their first hand experiences, but occasionally were based on what they had seen in the media and/or heard other equine stakeholders talk about. The issues identified fell into three subthemes: health related welfare problems, management practices that cause poor welfare and riding/training practices that cause poor welfare. Table 2 shows all of the welfare problems raised by the interviewees. All of the interviewees raised one or more problems within the health and management categories, whilst 23 of the 31 interviewees discussed problems within the riding/training category.

**Table 2:** Stakeholder perceptions of the welfare problems faced by horses in GB with numbers of interviewees raising each issue in parentheses.

Category	Total number of stakeholders discussing	Welfare problems raised by 10 or more interviewees (number of interviewees)	Welfare problems raised by fewer than 10 interviewees (number of interviewees)
Health	31	• Underweight (**20**) • Poor feet/foot care (**18**) • Overweight (**12**) • Internal parasites (**11**) • Laminitis (an inflammatory foot condition) (**10**)	• Dental problems (**9**) • Skin problems (**9**) • Lameness (**7**) • Metabolic diseases (**5**) • Musculoskeletal problems, including back problems (**4**) • Strangles (an infectious respiratory disease) (**3**) • Genetic defects (**3**) • Foot abscess (**2**) • Colic (abdominal pain) (**1**) • Dehydration (**1**) • Azotoria (a condition causing muscle cramp) (**1**)
Management	31	• Stabling horses 24 hours a day (**19**) • Underfeeding (**14**) • Inappropriate rugging (putting too many or too few rugs on a horse) (**13**) • Lack of water (**12**) • Over-feeding (**12**) • Social isolation (horses kept without visual and/or physical contact with other horses) (**10**)	• Incorrect feeding, including feeding high concentrate, low forage diets (**7**) • Tethering (**6**) • Inappropriate worming (**4**) • Not vaccinated (**3**) • Over-clipping (removing too much of the horses’ coat with clippers) (**2**) • Overstocking (**2**) • Fly grazing (grazing horses on someone else’s’ land without the owners’ permission) (**1**)
Riding/Training	23	• Inappropriate use of training aids (e.g. whips and spurs) (**13**) • Poorly fitting tack (**11**)	• Breaking in (training the horse to accept a rider) or ridden too young (**9**) • Rollkur (riding with the horses head and neck in a hyper-flexed position) (**5**) • Over-bitting (a bit is a metal bar placed in the horses’ mouth when riding to help control the horse. Riders may use overly ‘strong’ bits) (**4**) • Lack of clear aids (for example, not clearly asking the horse to ‘stop’ or ‘go forward’ resulting in confusion on the part of the horse) (**4**) • Heavy handed riding (**3**) • Unbalanced riders (**3**) • Over working (**3**) • Not warming horses up/cooling them down properly (**3**) • Rapping (a training technique used to encourage horses to jump higher and avoid knocking show jumps down. The pole is raised as the horse jumps over the fence so that the horse knocks its legs on the pole, thus encouraging the horse to make a greater effort the next time) (**3**)

#### Health related welfare problems

In relation to health, horses being underweight and foot problems were the two most commonly discussed issues, brought up by 20/31 and 18/31 interviewees respectively. Foot problems discussed included foot abscesses and horses with over grown hooves. Horses being overweight were discussed by 12 interviewees. Azoturia (a condition causing muscle cramp) and dehydration were each brought up as problems by single respondents.

#### Management related welfare problems

Nineteen out of the 31 interviews raised stabling 24 hours a day as a management practice they saw causing welfare problems in horses in GB. This is compared to only one who discussed fly grazing, the practice of grazing horses illegally on private or public land without the landowners permission, as a welfare issue.

#### Riding and training related welfare problems

Within the subtheme of riding and training, inappropriate use of training aids was raised by 13 interviewees. As described by the interviewees, inappropriate use of training aids included, for example, misuse of the whip and ‘forcing’ horses into a false outline using lunging aids or draw reins. Where the use of training aids were discussed by the interviewee it was not the use of the equipment per se that the interviewees felt caused welfare problems but the way it was used. Rapping (a training technique used to encourage horses to make a greater effort when jumping) was only discussed by three interviewees.

See Table 2 for all of the health, management and riding and training related welfare problems raised by the interviewees.

### Contexts in which welfare may be compromised

Stakeholders discussed contexts that they saw in GB in which they felt equine welfare was compromised. These were situations in which they believed welfare may be compromised as a result of a combination of risk factors, or welfare problems, rather than as a result of one single defined welfare problem or risk factor. The reasons why or manner in which they believed welfare may compromised in these contexts were discussed. See Table 3 for a full description of all the contexts raised by the interviewees. The most frequently discussed context (19/31 interviewees) was where horses are kept in unsuitable environments where, as perceived by the interviewees, a number of welfare risk factors may be present including: poached ground (grazing damaged by horses’ feet) which provides little nutritional value and discourages horses from lying down, ragwort in the grazing and other physical hazards, including poor fencing. Fifteen of the interviewees discussed how they saw welfare compromised when horses were ‘used’ inappropriately. Their descriptions of this context included situations where ridden horses are asked to do something they were unlikely to be capable of doing, for example an advanced dressage movement. The interviewees discussed a number of welfare challenges associated with this context including training techniques involving punishment and physical problems associated with the inappropriate work level. Fifteen of the interviewees believed that horse welfare in GB was compromised as a result of their behaviour being misunderstood. Stakeholders discussed how welfare may be compromised when ‘stress’ and pain behaviour was either not recognised or not correctly interpreted. The welfare problems associated with this were seen to be further compounded if, for example, the horse was punished for exhibiting unwanted behaviours. Fourteen of the 31 interviewees described how horse welfare in GB was compromised when horses changed hands, i.e. when they are bought and sold, or when they are moved from one home to another. Two believed that the moving process itself, and related change in environment and routine, was a welfare problem facing horses in GB. Others only discussed this context as a welfare problem for some horses in GB. In particular horses that had ‘problems’, for example horses that were lame or were exhibiting unwanted behaviours and/or had become of low financial or sentimental value, were seen to be particularly at risk.

**Table 3:** Stakeholder perceptions of the contexts in which welfare may be compromised, including the number who raised it and a description of the context as given by the stakeholders.

Context	Number of stakeholders raising	Welfare issues and welfare risk factors discussed by stakeholders in relation to the context
Horses kept in unsuitable environments	19	Physical hazards, poached ground, poor quality/no grazing, ragwort, small (taped off) paddocks, buildings/fencing in poor condition.
Inappropriate ‘use’	15	Riders trying to get their horses to do things which the horse is not physically capable of, horses asked to do things which they are not physically fit enough to do, administration of drugs to enhance the horses’ performance or enable the horse to be ridden.
Where behaviour is misunderstood	15	Pain and ‘stress’ behaviour may be misinterpreted or ignored and/or may be dealt with aggressively, e.g. through physical and/or verbal punishment.
Changing owners/Moving yards	14	Changes in routine and feeding linked with physical and mental welfare problems. Horses, particularly ‘low value’ or ‘problem horses’ can fall into the ‘wrong’ hands and may continuously change owners.
Abandonment	12	Horses may be truly abandoned, put out to pasture with little owner input or may be cared for at a livery yard, but are ‘abandoned’ by their owners.

Transportation	10	Long distance travel, associated exhaustion and dehydration, problems caused when loading where force is used to get horses on to the lorry.
Where horses don’t match expectations	8	Where horses are bought to perform a particular function, problems can occur when the horse can’t perform that function. Linked to horses becoming low value and being sold (see above).
Where euthanasia is delayed	8	Some people keep horses alive, usually for sentimental reasons, despite the horse having a poor quality of life.
Horse/rider/owner incompatible	8	People buy horses which they do not have the experience or ability to ride/manage. Discussed more in terms of human welfare (safety) but was also seen to have consequences for the horse for example if the horse gets dubbed as a ‘bad’ horse and becomes ‘low value’ (see above).
Where people own too many horses for their resources	6	People own more horses than they can afford/have time for, resulting in a range of welfare problems
Routine disrupted	6	Disrupted routine, routine based on the owner not horse, too rigid a routine, doing things which the horse is not used to.
Work/exercise unvaried	5	Horses may only do one type of work and therefore may be worked too intensively, may not be allowed to relax or may be bored.

### Sectors of horse use where welfare is compromised

Stakeholders were asked a direct question relating to the areas or sectors of the GB horse industry where they felt that welfare was particularly vulnerable to compromise. Interviewees were asked to explore their reasoning behind their opinions. The racing sector was suggested by 17/31 of interviewees as an area responsible for poor welfare. Over-breeding and intensive training were highlighted as particular problems within the sector. Furthermore, there was concern over what happened to horses when they could no longer race. Fifteen of the 31 stakeholders raised their belief that horses kept within the travelling community were vulnerable to poor welfare. Tethering and associated lack of water and food were frequently discussed as concerns. Mounted games, endurance, driving and eventing were discussed by one person each as sectors within GB where welfare was vulnerable to compromise. See Table 4 for a full description of the sectors raised by the interviewees and the welfare problems they associated with each of the sectors.

**Table 4:** Stakeholder perceptions of the sectors of horse use where welfare is compromised and the specific welfare issues they associated with them.

Sector/Horse Use	Number raising	Welfare issues and welfare risk factors linked by the stakeholders to the sectors
Racing	17	Over breeding, horses broken in and raced too young, injuries, intensive training, constant stabling and inappropriate diet. Concern was raised over what happens to race horses when they are no longer able to race.
Travellers	15	Tethering and associated lack of access to food/water, horses broken in too young and over worked on hard ground, horses do not receive routine health care.
Dressage	9	Horses are ‘forced’ to work ‘unnaturally’, e.g in Rollkur, training is physically and mentally intensive, training results in strain on limbs, horses are broken in too young, horses allowed limited access to pasture.
Livery Yards	8	Limited grazing and over stocking, horses housed and grazed separately due to owner fear of horse injury. Staff may give poor standards of care.
Leisure horses	7	Owners don’t always notice lameness or recognise the horses’ limitations, horses given limited access to pasture, euthanasia may be delayed for sentimental reasons.
Non-working horses	6	Horses get bored without the stimulation, non- working horses prone to being passed on as become ‘low value’, lameness may go unnoticed or untreated as the horse isn’t working.
Breeding	6	Brood mares not be well cared for, stallions kept in social isolation, weaning practices may cause physical and mental stress.
Sport (any)	5	Limited pasture access, long distance travel, physical demands, use of drugs to facilitate performance. There was also concern over what happens to horses when they can no longer be used for sport. One respondent thought it was ethically wrong to use horses for sport/competition.
Polo	5	Not fed during the day when working, lameness and injury, drug use to enhance performance, poor tack fitting, poor dental care, over-bitting, excessive whip use, horses vulnerable to contagious disease outbreak, ponies are in a poor condition at the end of the season.
Low level competition/riding club/pony club	5	Inconsistent work e.g. not exercised during the week then competed at the weekend, horses not be fit enough to do the work asked of them, owners more likely to get advice from the ‘wrong’ people.
Show jumping	4	Training techniques including rapping, strain put on joints, long distance travel, neurectomies carried out to continue use of horse.
Riding school/trekking centre	4	Horses may be over-worked or inconsistently worked, work may be ‘boring’, riders may lack ability, inexperienced/young people may be looking after the horses.
Semi-feral ponies	3	Overbreeding, under feeding during winter, ‘drifts’ (whereby semi-feral ponies are rounded up once a year to be counted, given veterinary treatment and selected for sale) are stressful for the ponies. Concerns were raised over where the ponies go when sold after drifts.
Showing	2	Training techniques, ponies often obese, over-rugging to keep coat thin, limited access to pasture, lack of variety in work, long distance travelling.
Hunting	2	Riders under the influence of alcohol, horses follow herd and are therefore at risk of injury.
Mounted games	1	Injuries to horses.
Endurance	1	Horses may be pushed too hard e.g. ridden over longer distances than they are fit enough for.
Eventing	1	Injuries during cross country phase, limited pasture access, social isolation.
Driving	1	Horses over worked on hard ground.

### Root Causes of Welfare Problems

Table 5 describes the ‘root causes’ (more fundamental, underlying causes that result in poor welfare in GB) as raised by stakeholders. The majority of stakeholders (27/31) believed that welfare problems in GB resulted from a lack of knowledge on the part of caregivers. Linked with this, 16/31 interviewees believed that poor advice seeking behaviour, for example asking people without the necessary knowledge for advice, resulted in welfare problems. Finances, in particular the lack of, was seen by 20/31 interviewees to result in poor welfare, for example where owners could not afford to call the vet out to their sick horse. The other root causes discussed by stakeholders in relation to poor equine welfare in GB were: Indiscriminate breeding, horses being viewed as commodities, welfare legislation, passport legislation and euthanasia costs.

**Table 5:** Stakeholder perceptions of the root causes of welfare problems in GB and ways in which the root causes were discussed by stakeholders.

Root cause	Number raising	Ways in which the root cause were discussed
Lack of Knowledge	27	Particularly linked to those who didn’t grow up with horses and first time owners. Areas where there was a perceived lack of knowledge included feeding, behaviour and lameness.
Finances	20	Lack of money was linked to horses not receiving vet treatment, dental care and foot care, horses not being insured, horses being kept in unsuitable environments and abandonment.
Advice seeking behaviour	16	Owners/caregivers not seeking advice, seeking and/or getting advice from people who don’t have the necessary knowledge to give good advice or not taking the advice given
Indiscriminate breeding	13	Horses bred without consideration of whether there is a market for them. Limited market results in them becoming ‘low value’.
Horses viewed as commodities	11	The relationship whereby horses are seen by their owners as commodities was linked to problems being caused when horse didn’t meet expectations/couldn’t do the job the owner wants them to do.
Welfare legislation	6	Legislation, for example the Animal Welfare Act 2007, is inadequate and/or poorly enforced. The welfare charities are only able to deal with the worst of the welfare problems.
Passport legislation	6	Legislation is insufficiently enforced and does not effectively link horses to owners. ‘Signing out’ of passports when horses are given restricted medications results in horses not being able to go through abattoirs, thus removing their slaughter value.
Euthanasia costs	2	As well as above, the cost of euthanasia was seen as a problem as there is no ‘free’ way to get rid of unwanted horses that cannot enter the human food chain.

### Associations between the role of the interviewees and their responses

The 2-sided Fischer’s exact tests revealed a number of statistically significant associations between the role category of the interviewees and their responses.

Interviewees that fell into the charity category (n = 6, see Table 1) were significantly more likely to raise the following welfare problems than those in the other categories: high body condition score (p = 0.022), overfeeding (p = 0.022), lack of water (p = 0.022), tethering (p = 0.006), not vaccinating (p = 0.004), contexts where owners have too many horses for their resources (p = 0.069) and travellers as a sector where horse welfare was compromised (p = 0.007).

Interviewees falling into the health category (n = 8, see Table 1) made significantly less references to underfeeding as a welfare problem (p = 0.045) and lack of knowledge as a root cause of poor welfare (p = 0.043) than those in the other categories.

Riders/trainers (n = 9, see Table 1) raised contexts where horses were incompatible with their owner in relation to poor welfare more than those not in the rider/trainer category (p = 0.027). They also discussed commodification of the horse as a root cause of poor welfare more than the other interviewees (p = 0.03). Contexts where horses are kept in unsuitable environments were raised significantly fewer times by those in the riders/trainer category than those in the other three categories (p = 0.012).

## Discussion

This study used a qualitative approach to investigate equine stakeholders’ perceptions of the welfare problems experienced by horses in GB and gives insight into the welfare problems potentially faced by horses, the contexts in which welfare may be compromised and the possible root causes of poor equine welfare in GB. The semi-structured interview approach insured that the research frame was not limited by the researchers’ preconceptions of the welfare problems which exist. Kauppinen et al [18] used qualitative interviews as a preliminary research step when investigating farmers’ attitudes towards improving animal welfare. By taking this approach they were able to focus subsequent research on the relevant issues and also identified new themes which had not come out in previous studies. Similarly, in this study, a number of equine welfare problems were identified which have, as yet, not undergone surveillance in GB. As such these findings may be used to direct further research and welfare improvement interventions.

### Health related welfare problems

All of the stakeholders interviewed discussed welfare problems which fell into the health category. This is unsurprising as welfare has historically been seen as synonymous with health and physical functioning [22]. Issues discussed in this current study as affecting horses in GB included horses being under weight, horses being overweight, poor feet/foot care, laminitis and colic. Equine health has undergone some surveillance across the GB equine industry. For example The National Equine Health Survey (NEHS) is run every year by the Blue Cross and gathers data on the health status of horses, ponies, donkeys and mules in the UK [23]. In addition studies have been conducted investigating the prevalence and causes of specific health issues in GB including laminitis [5] and obesity [24], and health issues affecting particular groups of horses including geriatric horses [25], veterinary registered horses [26] and tethered and free range horses [8,9]. Based on the findings from the current study there is no evidence that there are specific health issues within the GB equine population which have gone unrecognised by researchers, although a more comprehensive understanding of nationwide prevalence of many health issues is needed. Whilst there has been research into the potential welfare consequences of some of the health issues raised by the interviewees for example laminitis [27], and back problems [28], further research is needed to understand the effects of many of these health conditions, for example how these health issues impact on the affective state of horses.

The findings from the 2014 NEHS [29] suggests a relatively high prevalence of sarcoids and allergic respiratory disease within the UK equine population (5.6% and 7.1% respectively) and Ward et al [30] reported a prevalence of gastric ulcers in GB domesticated horses of 60.8%. Ireland et al [26] reported the presence of a number of health issues, including respiratory problems, sarcoids, eye problems and cardiac problems, within the GB horse population. None of these conditions were raised by the interviewees possibly because they had no experience of these in the horses they came into contact with or because they did not perceive them to be a welfare problem. Their lack of inclusion may also be a reflection of the methodology and these issues may have not been at the forefront of the interviewees’ minds at the time of the interview.

### Management practices causing poor welfare in GB

Welfare problems associated with the management of horses were discussed by all stakeholders suggesting that they also linked some of the equine management practices utilised in GB to poor horse welfare. Management practices leading to welfare problems raised by the interviewees in this current study included stabling horses 24 hours a day and keeping horses in social isolation. The current available literature supports the interviewees’ views that these practices occur within GB. For example, Hockenhull and Creighton [31] reported that a small proportion of UK leisure horses (three percent) had been given no access to pasture in the week prior to their carers completing an on-line questionnaire. Nine percent had no other horses present within their environment with which to freely interact. There is also evidence to support our interviewees’ perceptions that these practices can impact negatively on welfare. For example, Hockenhull and Creighton [31] found that when compared to stabling for 1–4 hours a day, stabling horses for 13 to 16 was associated with aggressive behaviour and handling issues and spending 21–24 hours stabled was associated with abnormal oral or ingestive behaviour. Lesimple et al [32] found that horses that spent between 13 and 24 hours in single stables were more active when released into an arena than horses kept in paddocks whilst Christensen et al [33] found that singly housed stallions engaged in more social grooming and play behaviour than group housed stallions when subsequently allowed to freely interact with other stallions. These observed behaviours, seen in horses kept in confinement and/or social isolation suggest that welfare may be compromised when horses are not provided with access to pasture and/or social contact. As such, the current literature supports the interviewees’ perceptions that some horse in GB may be experiencing suboptimal welfare as a result of stabling and/or limited social contact.

The interviewees in the current study also felt that feeding practices were a cause of poor welfare for horse in GB. Giles et al [24] found that feeding practices undertaken by equine caregivers in England were associated with the presence/absence of obesity in outdoor living whilst Wylie et al [5] found that new access to grass in the previous 4 weeks week prior to owners completing their survey were both associated with an increased risk of developing laminitis. As such, there is some research to support our interviewees’ perception that some horses in GB may experience poor welfare as a result of feeding practices. The interviewees in the current study highlighted ‘incorrect’ feeding and in particular the provision of low forage diets as a welfare challenge facing horses in GB. Current research supports the view that the amount and type of forage provided for horses has welfare consequences. For example, the potential welfare benefits of providing foraging opportunities [34] and a mixed forage diet [35] have been noted and increasing time at pasture has been found to reduce the risk of colic recurrence [36]. Wylie et al [37] reported that the majority (92.1%) of GB veterinary registered horses have access to grazing for at least part of the day and many (82.6%) receive additional forage in the form of hay or haylage, especially during the winter months. 86.1% also receive additional concentrate feeds. Further research is likely to be beneficial to understand more about the feeding practices experienced by GB horses and the ways in which these practices impact on the welfare of horses.

Some potential management related welfare problems highlighted by the interviewees in this current study have not undergone surveillance in GB. For example, inappropriate rugging which was discussed by nearly half (13) of the respondents. Mejdell et al [38] studied horse rug preference by training horses to select a ‘rug off’, ‘rug on’ or ‘stay the same’ symbol based on their preference and found that the choices made varied between horses and was influenced by the weather. Little is known about the prevalence of rug use in GB, how rug use interacts with welfare measures such as thermal comfort and how owner choice may differ from horse choice suggesting an area where further research may be beneficial.

When discussing breeding practices in GB, our interviewees brought up weaning methods as a welfare challenge facing GB horses. Current research suggests that equine welfare may be affected as a result of weaning methods including post weaning housing and social conditions [39, 40] and post weaning feeding [40]. Further to this, there is some evidence that weaning practices in GB may be challenging the welfare of horses in GB. Greening and Febery [41] found that on 81% of UK studs foals were weaned abruptly and that small studs tended to house weaned foals in pairs or individually. Waters et al [40] found links between weaning practices and the development of stereotypic behaviour in horses in GB. As noted by Waran et al [42], there is a need for a greater understanding of what constitutes ‘best practice’ in relation to weaning. When used in conjunction with further knowledge of current practice in GB, this will help us better understand the welfare impact of weaning practices for horses in GB thus informing routes to improvement.

### Welfare problems associated with riding and training

In comparison to the number that brought up health and management related welfare issues, fewer interviewees (23/31) brought up welfare problems associated with riding and training. This was possibly because some stakeholders did not perceive riding and training to relate to welfare or because riding and training related welfare problems are unrecognised by some stakeholders. It may also reflect the fact that a proportion of the interviewees were not directly involved in the riding and training of horses, although there was not a statistical association between the role category and whether or not riding/training related welfare problems were discussed.

Hockenhull and Creighton [43] investigated the riding and training practices that are experienced by UK leisure horses as potential risk factors for ridden behaviour problems. In a survey of 1326 horses Hockenhull and Creighton [43] found that an increased risk of ridden behaviour problems was associated with specific types of tack, the use of particular training aids and absence of saddle fitting checks. These findings could indicate that the equipment used by riders on their horses may result in reduced welfare. However, some equipment used by riders may be indicative of pre-existing conflict behaviours, and as such the training equipment used may be an indicator of a welfare problem rather than a cause persay. It should be noted that the interviewees in the current study particularly referred to the way in which training equipment was used, i.e. that welfare problems occurred specifically as a result of ‘inappropriate’ use. Whilst the study by Hockenhull and Creighton [43] gives insight into the associations between training equipment and behaviour as a possible indicator of horse welfare, they did not investigate the ways in which the equipment was used, pointing to an area where further research is needed.

Several other welfare challenges relating to riding and training that were raised by our interviewees have undergone some scientific investigation. Our interviewees felt that poorly fitting tack posed a welfare challenge to GB horses. Hockenhull and Creighton [43] reported an association between saddle fitting and behaviour problems suggesting a possible link between saddle fit and horse welfare in GB. Lesimple et al [28] reported associations between rider posture (e.g. balance), back problems in horses and their attitude to work supporting our interviewees’ views that unbalanced riders may pose a welfare challenge. Riding Rollkur was discussed by 5 interviewees as a cause of poor equine welfare and this view is supported by the current literature [44–48]. Finally the potential negative consequences of bit use has been investigated [49], offering possible support for the interviewees views that over bitting causes poor welfare in horses in GB.

There is still the need for further research to explore, in particular, the specifics of riding and training practices experienced by GB horses and their prevalence to fully understand the riding and training welfare problems faced by horses in GB.

### Contexts associated with poor welfare

Whilst individual, discrete welfare problems, for example laminitis, were discussed by the interviewees in this study, they more frequently discussed broader contexts in which welfare may be compromised. These included contexts where horses are kept in unsuitable environments, where horses are ‘used’ inappropriately and where horses do not match the expectations of their owner/rider. Contexts where horses are transported were raised by 10 of the interviewees as a potential cause of poor welfare for horses in GB. Wylie et al [37] reported that 22.5% of the UK veterinary registered population had been transported in the week prior to their owners completing the researchers’ survey. There has been some research into the potential consequences of transportation on horses (see, for example Padalino et al [50] and Schmidt et al [51]) offering some support to the perception that transport may be a cause of poor welfare in horses in GB. Furthermore, [52] found that 8.8% of discharged equine in-patients at a veterinary practice in the UK were difficult to load, an indicator that some horses in GB may experience poor welfare as a result of transportation. More knowledge is needed about the numbers of horses being transported, the conditions and the welfare consequences of transport practices engaged in with GB to fully appreciate the welfare consequences of transport for horses in GB.

One theme that frequently came up in the interviews related to the potential welfare challenge faced by horses when they changed hands, for example when they were bought and sold. This context was linked to other identified contexts, specifically where the horse does not match expectations of, or is incompatible with, its owner/rider. Hotchkiss et al [53] found that the average length that a horse is owned by the same owner is 4.9 years, supported by Wylie et al [37] who found that average ownership length was five years. Change of ownership may have a number of consequences for the horse including change in management regime, training regime, environment and social environment. Horses do not appear to habituate to regular social regroupings [54] suggesting that even without considering associated changes in environment and routine, changes in ownership, or changes in home environment within ownerships, are likely to be stressful for the horse. In order to advance our understanding of equine welfare problems facing horses in GB there is a need to fully explore the number of horses that are affected by all of the contexts raised by the interviewees and the specific welfare consequences for horses in GB.

### Areas of horse use/disciplines where welfare is compromised

When asked to discuss the areas of horse use or specific disciplines in which welfare was particularly compromised there was a tendency for stakeholders to bring up the same areas of concern and the racing industry and travelling community were discussed by 17 and 15 interviewees respectively. These perceptions may be a reflection of media influence, which emphasises welfare problems in some contexts more than others. For example, there has been extensive media coverage of abandonment of large numbers of travellers’ horses and of deaths which occur during the Grand National, one of the main national hunt races in GB. The emphasis of those interviewed may also be a reflection of the sample, which underrepresented the areas which came under the greatest amount of scrutiny. As reflected in this study, it is common to think of the horse industry as divided in terms of disciplines and/or horse uses and to suggest that there are welfare problems typical of, and even unique to, these disciplines. There is some evidence to suggest that the type of work engaged in by horses may have specific welfare consequences, and associations have been found between type of work done by an individual horse and the health related problems they experience [55]. Furthermore associations have been found between the likelihood of an individual horse expressing stereotypic behaviour and the type of work that they do [56]. Only through comprehensive assessment of welfare across the population can the interviewees’ views that horses in GB experience welfare problems as a result of the sector in which they are used this be confirmed or refuted. This is of particular importance as Horseman et al [21] found that there was a tendency for the interviewees to blame poor welfare on ‘others’ whilst sometimes overlooking and/or downplaying evidence of poor welfare in their own contexts.

### Underlying ‘root causes’ of poor welfare

Lack of knowledge and poor advice seeking behaviour as root causes of the welfare problems experienced by horses were discussed by 27 and 16 interviewees respectively, suggesting that these are perceived to be the predominant causes of poor welfare in GB. Hemsworth et al [57] state that lack of knowledge, on the part of horse owners, is one horse owner factor associated with poor welfare whilst the UK equine welfare charities have invested resources into their education strategies indicating that they also perceive this to be an important factor in equine welfare. However, scientifically studying how caregiver knowledge impacts on welfare is highly problematic, as there is no gold standard way of assessing it. Furthermore, lack of knowledge or awareness may not be the sole reason behind examples of poor welfare. Visser et al [58] found that horse owners that had knowledge about management practices that support positive equine welfare did not always implement those management practices. Horseman et al [21] suggests that practical constraints may be one reason why knowledge of best practice in relation to equine welfare does not always result in behaviour on the part of the owner to implement those practices. Furthermore, Horseman et al [19] found that delayed treatment of lame dairy cows had less to do with farmers’ awareness of lame cows and their knowledge of treatment methods than with their perceptions of the value of prompt treatment and practical constraints and barriers associated with the complexity of running a commercial farm. Whilst it may prove important to understand where specific knowledge gaps exist in relation to equine welfare, those interested in improving equine welfare should recognise that simply sharing knowledge may be insufficient for equine welfare improvements to occur and that understanding how and why people manage their horses in the way they do may also be important.

The statistical associations between the interviewees’ roles and the welfare problems that they discussed suggest that some biases may exist either in the welfare problems that individuals see or in what they perceive to be welfare problems. For example, charity workers may see more cases of high body condition score and overfeeding through their work but also may have more of an awareness of these as welfare problems. Similarly rider and trainers may not have first- hand experience of horses being kept in, what they would perceive to be, poor environments, due to the contexts in which they work. It is also possible that they do not recognise the welfare compromises caused by some of the environments they commonly see horses being kept in (see [21] for full discussion).

### Limitations of the study

The recruitment method adopted could not be said to provide a statistically representative sample from each of the identified stakeholder categories and biases may have been introduced, for example through inadvertent recruitment of people with similar perceptions. In addition, data regarding the age, gender and years of equine experience of the interviewees was not consistently collected and/or not reported as part of this study, limiting our understanding of the study population.

The interview approach used in this current study, whereby the interviewees were not prompted to discuss particular issues, may have resulted in individual interviewees discussing a limited range of welfare problems, constrained for example by what was at the fore front of their mind at the time of the interview.

The methodology revealed that the word ‘welfare’ was interpreted in different ways by the interviewees (for full discussion see [21]) and this may also have affected the issues discussed by participants as ‘welfare’ problems. As a result the researchers had to use a degree of interpretation when deciding which issues raised by the participants were included in the current report of ‘welfare’ problems.

Finally, it is recognised that animal caregivers may not always accurately assess the welfare of animals in their care as a result of, for example, over exposure to particular welfare indicators [59]. Furthermore, observers may focus their attention on behavioural indicators that do not provide accurate information about the affective state of the animal [60].

Taking these limitations into account, the findings of this current study should not be considered an exhaustive representation the actual welfare problems facing horses in GB or even of stakeholder perceptions of these. Care should also be taken when interpreting the findings when considering the number of respondents discussing each issue as an indication of the scale of the problem.

Never the less, in light of the limited surveillance of equine welfare in GB this research can be used to inform future endeavours to improve equine welfare in GB

## Conclusions

The findings from this research give an indication of the range of welfare problems facing horses in GB and stakeholders’ perceptions of the welfare of horses. This is of particular value in light of the limited empirical data available regarding equine welfare in GB. Those with first- hand knowledge of the problems facing horses may be well placed to highlight issues not considered by researchers. The diversity of the issues discussed in this study demonstrates the value of the methodological approach whereby a broad cross-section of stakeholders were interviewed and given the opportunity to freely discuss their perceptions of welfare not limited by any preconceptions held by the researchers. The large number of under-researched issues identified indicates that, in light of limited resources, further qualitative research may be beneficial to prioritise issues identified to target research and interventions. It will be useful to continually reflect on how perceptions of welfare align with the empirical evidence when thinking about welfare-improving strategies.
